# Charge Trap and Oxygen Barrier Engineering in Voltage-Stabilizing Grafted Silicone Rubber via Multiscale Molecular Simulations

**DOI:** 10.3390/polym18070780

**Published:** 2026-03-24

**Authors:** Jing Sun, Xindong Zhao, Zhongyuan Li

**Affiliations:** 1 Heilongjiang Institute of Technology, Harbin 150050, China; 2 School of Electrical and Electronic Engineering, Harbin University of Science and Technology, Harbin 150080, China; 3 Electric Power Research Institute, State Grid Heilongjiang Electric Power Co., Ltd., Harbin 150030, China

**Keywords:** addition-cure silicone rubber, chemical graft, charge trap, molecular simulation

## Abstract

The present theoretical study proposes and unravels chemical graft modification using a novel voltage stabilizer (3-amino-5-chlorophenyl 3-fluorophenyl methanone, ACFM) to ameliorate electrical insulation performance, oxygen-resistant characteristics, and thermal stability of addition-cure silicone rubber (SiR) used for cable accessory insulation in power transmission systems. First-principles calculations demonstrate that chemically grafted ACFM introduces shallow hole and electron traps into addition-cure SiR macromolecules to respectively impede hole transport and restrict hot electron production. Through molecular dynamics and Monte Carlo simulation, the chemically grafted ACFM is verified to enhance chain segment coalescence and decrease oxygen compatibility of addition-cure SiR macromolecules due to its higher dipole moment, leading to a reduction in oxygen permeation and improvement in thermal stability of the SiR crosslinked material. It is indicated from first-principles oxidation reaction paths that chemical grafting ACFM contributes positively to the oxidative stability of addition-cure SiR. The improved abilities of charge trapping and withstanding high temperatures together with enhanced resistance to both oxygen infiltration and oxidation of the addition-cure SiR material, as unraveled on a molecular scale in this research, open an avenue for developing advanced polymer dielectrics applied in harsh environments.

## 1. Introduction

High-voltage cable accessories are the most vulnerable links in modern power grids, where insulation materials must simultaneously withstand intense electric fields, transient thermal surges and ambient oxygen/moisture attacks over decades [1,2,3]. Addition-cure silicone rubber (SiR) is the incumbent solution because of its intrinsic flexibility, high dielectric strength and hydrophobic surface [4,5]. Nevertheless, the onset of free-volume-mediated avalanche injection, oxygen-induced chain scission and thermal oxidative crosslinking ultimately limit its long-term reliability [6,7,8]. Over the last decade, molecular engineering of polymer dielectrics has shifted from passive nanofiller blending to active chemical functionalization, in which small amounts of precisely designed polar molecules are grafted onto the backbone to tailor trap landscapes and transport barriers [9,10,11]. For SiR, vinyl-containing siloxanes allow for efficient hydrosilylation grafting under mild conditions, offering a versatile platform for introducing voltage stabilizers, antioxidant moieties or charge-blocking segments [12,13].

Experimental evidence reveals that shallow traps (0.3–0.8 eV) accelerate charge de-trapping and local Joule heating, whereas deep traps (1.0–1.6 eV) can store injected carriers and homogenize the electric field in cable accessories [14,15]. Grafting π-conjugated or electron-withdrawing groups is an established method to improve trap distribution, while the quantitative depth, density and spatial location required for SiR remain elusive. First-principles calculations have recently enabled trap-level prediction with sub 0.1 eV accuracy, but most studies focus on polyethylene, polyimide or epoxy resin [16,17,18], lacking sufficient electronic evidence for chemically modified SiR [19].

Oxygen diffusivity and permeation follow a solution–diffusion mechanism, whereby oxygen molecules first dissolve into the free volume and then react with methylene or methyl groups of the polymer backbone via radical chain reactions [20]. Reducing the solubility coefficient (S) and the diffusion coefficient (D) simultaneously is therefore essential. Previous work employed bulky side groups or fluorinated segments to condense the free volume, generally compromising cure kinetics and mechanical compliance [21]. Grafting polar molecules to generate dipole–dipole crosslinks offers a filler-free means of densifying silicone segmental packing, yet the influence of graft architecture on S, D or the oxidation barrier has not been quantitatively correlated [22].

Cable accessories experience repeated load cycles that impose ∆*T* ≈ 150 °C within minutes. Local hot spots trigger Si-O/Si-C bond homolysis and rearrangement, leading to stiffness increase and crack initiation [23]. While bulk modifications such as MQ (methyl-phenyl-vinyl) silicone resins improve thermal stability, they concurrently incur phase segregation and escalate material costs [24]. Grafting heat-resistant hetero-aromatics or conjugated ketones can potentially scavenge thermal radicals and dissipate vibrational energy via internal conversion, but the underlying mechanisms remain speculative without atomistic insights [25].

Molecular dynamics simulations are indispensable in polymer sciences to elucidate structure–dynamics–mechanics relationships of elastomers under disparate loads, reveal the thermodynamic origin of contact electrification between dielectric polymers, optimize cable-grade high-voltage insulators, underpin the mechanistic optimization of multifunctional macromaterials, and guide the supramolecular design of nanocomposite networks [26,27,28,29,30]. In parallel, first-principles calculations provide electronic-scale insights into rational dielectric engineering, accelerating materials discovery when merged with data-centric workflows, accurately dissecting thermal-transport limits in polymer dielectrics, and identifying functional groups at polymer/metal interfaces that confer strong adhesion to metallic electrodes in solid polymer electrolytes [31,32,33].

Voltage stabilizers, encompassing acetophenone derivatives, triphenylmethane, naphthalene-based compounds, and aryl carbonyl functionalities, have been predominantly employed in the chemical grafting modification of crosslinked polyethylene (XLPE) cable insulation [34,35], ethylen-propylen-diene monomer (EPDM) or silicone rubber for cable accessories [25,36,37,38,39], and epoxy resin formulations for insulator casting [40], with the objective of enhancing their electrical insulation performance. It has been experimentally validated that these grafted moieties can effectively suppress space charge accumulation and electrical tree propagation through the introduction of deep charge traps, thereby substantially elevating the dielectric breakdown strength of polymer dielectrics [41,42]. While certain voltage stabilizers demonstrate favorable compatibility with low-polarity polymers such as silicone rubber when employed as chemical-grafting agents, the covalent functionalization of silicone rubber with voltage stabilizers for dielectric performance enhancement remains largely unexplored. Building on these developments, the present work introduces a multifunctional voltage stabilizer that can be facilely grafted onto addition-cure SiR. Leveraging molecular simulation techniques, particularly by molecular dynamics simulations and first-principles calculations, the present study explores the voltage stabilizer modification on addition-cure SiR for ameliorating electrical-insulation-relevant performances. The fundamental mechanism governing the interaction between the grafted agent and the host SiR is unraveled for obtaining molecular-level insights into charge trapping, oxidative stability (reaction path), oxygen infiltration (diffusion and uptake), and thermal stability of addition-cure SiR, which offers valuable guidance to design and develop advanced SiR dielectrics applied for cable accessory or encapsulation insulation.

## 2. Theoretical Model and Calculation Methodology

Currently, novel voltage stabilizers employed to enhance the dielectric performance of polymeric insulating materials utilized in power transmission systems, including XLPE cable compounds, EPDM or silicone rubber cable accessories, and epoxy resin insulators, primarily comprise 4-vinyloxy phenylethanone, 4-acrylicoxy 2-hydroxydiphenone, and 2-amino-5-chlorophenyl 2-fluorophenyl methanone (2-ACFM) [35,37,40]. Although 2-ACFM possesses more effective multiphenyl strongly polar functional groups among these voltage stabilizers, the chemically reactive site for grafting modification (amino group) in 2-ACFM molecules is adjacent to a carbonyl-bridged diphenyl structure, resulting in significant steric hindrance and causing the grafting reaction of 2-ACFM molecules to be kinetically unfavorable. Consequently, the present study proposes an isomeric analogue of 2-ACFM, internationally standardized as 3-amino-5-chlorophenyl 3-fluorophenyl methanone (ACFM), to enhance the electrical insulation performance of addition-cure SiR via chemical grafting. It should be noted that the primary amino group in ACFM may poison platinum-based catalysts used in addition curing. Practical implementation would require protecting group strategies or advanced catalyst formulations, which are beyond the scope of this computational proof-of-concept study.

**Figure 1:**
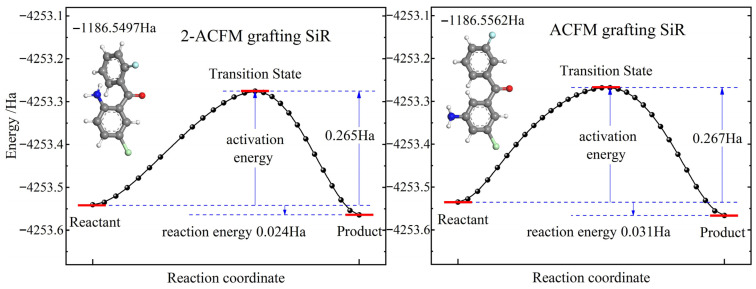
Chemical reaction paths (minimum energy paths) of grafting 2-ACFM and ACFM respectively onto the vinylsiloxane of addition-cure SiR, inserted with their molecular models and ground-state total energy values. The white, gray, blue, red, light blue, and light green spheres symbolize hydrogen, carbon, nitrogen, oxygen, fluorine, and chlorine bonding atoms respectively.

To further elucidate the chemical kinetics and validate the enhanced reactivity of ACFM, the minimum energy paths for grafting 2-ACFM and ACFM respectively onto the vinyl of addition-cure SiR are calculated by the first-principles method (methodological details are provided in Section 2.1 and Section 2.3), as shown in Figure 1. Comparative analysis of the two isomers reveals that ACFM exhibits a lower ground-state total energy (−1186.5562 Ha vs. −1186.5497 Ha of 2-ACFM) and releases a greater reaction energy upon grafting onto SiR (0.0312 Ha vs. 0.0236 Ha for 2-ACFM) despite both isomers overcoming similar reaction energy barriers (activation energy, ~0.266 Ha). The grafting of ACFM forms a graft product with lower total and binding energies (−4253.5661 Ha and −18.7676 Ha respectively compared to −4253.5641 Ha and −18.7656 Ha for 2-ACFM). Relative to 2-ACFM, ACFM demonstrates a more stable molecular configuration (conferring higher chemical synthesis efficiency and yield) and facilitates more facile grafting reactions with vinyl groups of addition-cure SiR through its chemically reactive amino group, while retaining the phenyl polar moieties (chlorophenyl, fluorophenyl, and benzoyl) essential for suppressing charge carrier transport and electron impact ionization.

### 2.1. Macromolecular Electronics and Thermodynamics

**Figure 2:**
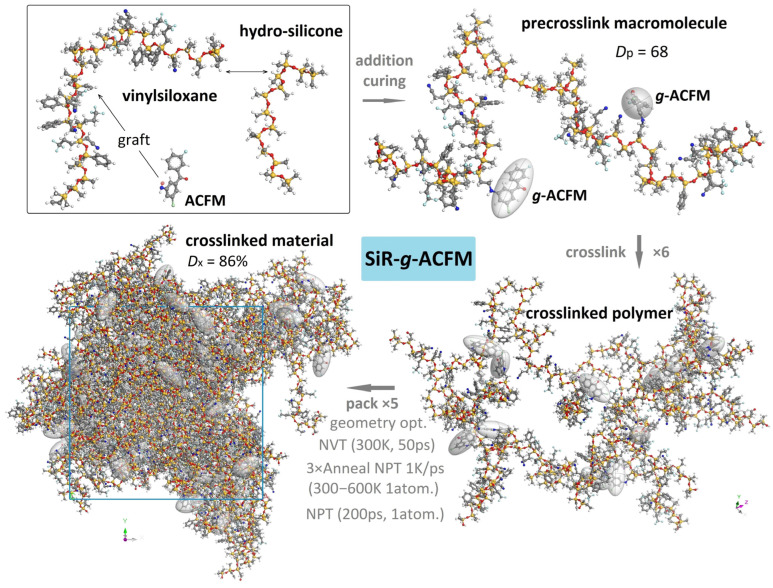
Schematic process for modeling macromolecules and materials of addition-cure SiR grafted with ACFM to evaluate charge traps, oxidative stability, O_2_ infiltration, thermal stability by first-principles calculations as well as MC and MD simulations. The white, gray, blue, red, light blue, orange and light green spheres symbolize hydrogen, carbon, nitrogen, oxygen, fluorine, silicon and chlorine bonding atoms respectively.

The target polymer system is an addition-cure SiR whose network is chemically modified by grafting ACFM. The SiR and ACFM grafted SiR (SiR-*g*-ACFM) precrosslink macromolecules composed of vinylsiloxane rubber (raw SiR) copolymerized with hydro-silicone oil (addition vulcanizing agent) are firstly built up using a rotational isomeric state (RIS) model and geometrically optimized to calculate electron states, polarizability, electrostatic potential, and molecular vibration by a density-functional-based (first-principles) tight binding method (Table 1), as implemented within DFTB code of the Materials Studio (MS) software package (BIOVIA v23.1.0.3829, Accelrys Inc., San Diego, CA, USA). Given the relatively high chemical activity of the vinyls (-C=C-) residing in vinylsiloxane constituent of addition-cure SiR, the -C=C- alongside the -Si-O- backbone is chosen as the grafting site for modeling the SiR-*g*-ACFM macromolecule. Meanwhile, the grafting ACFM molecule with its nitrogen–hydrogen bond (-NH) as the grafting point chemically connects and saturates a -C=C- randomly located in the SiR macromolecule, which approaches a grafting content of 6.2 wt%. Thermodynamic compatibility between SiR or SiR-*g*-ACFM precrosslink macromolecule and oxygen molecule (O_2_), quantified by mixing energy and interaction parameters, is investigated via configurational-bias Monte Carlo simulations in the isothermal–isobaric ensemble combined with the Flory–Huggins model (the specifications are listed in Table 1), employing the Widom test particle insertion method to evaluate the excess chemical potential of O_2_ in the polymer matrix [43,44,45]. According to the crosslinked SiR materials with high transparency and low viscosity applied for cable accessory and electronic packaging, the modeled SiR and SiR-*g*-ACFM precrosslink macromolecules approach 68 degrees of polymerization (*D*p, molar mass = 7530 g/mol and 8030 g/mol respectively) as determined by the number of backbone silicon–oxygen bonds, which are then crosslinked (×6) by their vinyls for constructing the crosslinked polymer-molecule models with molecular weights of 45,185 g/mol and 48,181 g/mol respectively, as shown in Figure 2.

Normal vibrational modes (intrinsic vibrational frequencies and intensities) of molecular chains are calculated by constructing the Hessian matrix via two-point finite-difference analysis of forces for thermodynamic property analysis [51]. For a molecule (or any finite atomic system), the Hessian matrix element *H*_i,j_ is defined as the second partial derivative of the total energy *E* with respect to Cartesian coordinates q_i_ and q_j_, i.e., *H*_i,j_ = *∂*^2^*E*/*∂*q_i_*∂*q_j_. Division of *H*_i,j_ by the square root of the corresponding atomic masses yields the mass-weighted Hessian matrix ***F***. Under the harmonic approximation, the vibrational frequencies correspond to the square roots of the eigenvalues of ***F***, with the associated eigenvectors *F*’ representing the normal vibrational modes. The vibrational intensities are determined by the polarizability tensor ***A*** of all atoms in the system (defined as the second partial derivative of the total energy with respect to Cartesian coordinates and dipole moment, commonly referred to as Born effective charges in solid-state calculations). The intensity of normal mode i is given by the squared sum of all transition moments for that mode: *I*_i_ = (Σ_j,k_*F*’_i,j_*A*_j,k_)^2^.

### 2.2. Condensed Matter Model and O_2_ Infiltration

Condensed matter models of SiR and SiR-*g*-ACFM materials are established through packing five SiR or SiR-*g*-ACFM crosslinked polymer molecules with a initial density of 1.0 g/cm^3^ and a crosslink degree (*D*x) of 86% into an amorphous supercell by MC molecular simulations, further being relaxed to thermodynamic equilibrium under barostatic atmosphere pressure at diverse temperatures of 300–600 K through MD simulations under a polymer consistent force field (PCFF), as illustrated in Figure 2. The delicate schemes and specifications in the MC and MD simulations of the SiR and SiR-*g*-ACFM materials in this study, as implemented within the MS package, are similar to the reports in references [19,52].

Material structural characteristics and MD statistical properties, including fractional free volume (FFV), isobaric heat capacity (*C*_p_), cohesive energy density (CED), and ensemble-averaged atomic or O_2_ self-diffusion coefficients, are calculated through statistical analyses of NPT MD-simulated condensed-phase models at thermodynamic equilibrium: (1) FFV is evaluated using the Atom Volume Surfaces tool in MS Forcite by calculating the occupied volume (*V*_O_) and free volume (*V*_F_) based on the van der Waals surfaces of molecular chains within the modeled polymer material, as determined by the formula FFV = *V*_F_/(*V*_O_ + *V*_F_); (2) *C*_p_ is derived from the partial derivative of enthalpy (*H*) with respect to temperature (*T*) at constant pressure (*P*), expressed as *C*_p_ = (*∂H*/∂*T*)*_P_*, through NPT MD simulations wherein internal energy (*U*), volume (*V*), *T*, and *P* are extracted to calculate *H* = *U* + *PV* as a function of *T*; (3) CED is calculated as the sum of van der Waals and electrostatic energy densities, defined as the total non-bonded interaction energy divided by the simulation cell volume, to quantify intermolecular cohesion and mechanical integrity; (4) the self-diffusion coefficients of selected atoms or molecules are determined from their mean squared displacement (MSD) as a function of time, obtained from coordinate trajectories in the MD simulations, with linear fitting of the MSD–time relationship and division of the resulting slope by a factor of 6 (accounting for three-dimensional bidirectional random motion) yielding the isotropic self-diffusion coefficient for the specific atomic or molecular species.

### 2.3. Oxidative Reaction Path

To evaluate the impact of chemical grafting on oxidative stability of addition-cure SiR, the energy barriers and released energies for both the addition-cure SiR constituents (hydro-silicone and vinylsiloxane with their terminals being hydrogenated, representing the polymerized states) and the ACFM molecule grafting on SiR (the molecular fragment containing the grafted ACFM in SiR-*g*-ACFM) reacting with O_2_ are calculated by a density-functional-based tight binding method (Table 1). The protocol proceeds in three stages: (i) reactant and product of the SiR constituents and the grafting ACFM molecule with O_2_ are geometrically optimized to their ground state; (ii) the minimum energy path of oxidation reaction is calculated by transition state searching (constructing a series of intermediate reacting structures as a reaction coordinate) with the quadratic synchronous transit (QST) method; (iii) the energy barrier or released energy is determined by the energy difference between reactant and transition state or product along the reaction path.

## 3. Results and Discussion

### 3.1. Charge Traps and Polarizability Introduced by Grafting ACFM

**Figure 3:**
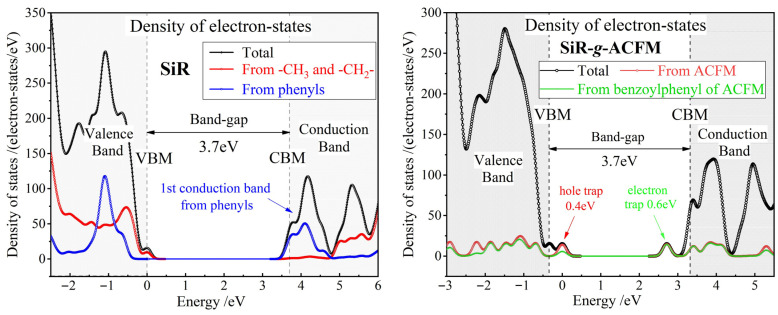
Density of electron states (Gaussian smearing = 0.1 eV) from energetic spectra of molecular orbitals for the SiR (**left panel**) and SiR-*g*-ACFM (**right panel**) precrosslink macromolecules. The highest occupied molecular orbital is referenced as energy zero.

First-principles energetic density of electron states indicate that the ACFM graft can efficiently introduce electron/hole traps in depths of 0.6/0.4 eV in SiR precrosslink macromolecules, as respectively shown by the unoccupied/occupied electron-bound states just below/above the conduction band minimum (CBM)/valence band maximum (VBM) of the SiR-*g*-ACFM precrosslink macromolecule in Figure 3. This kind of attribute in electronic properties indicates that the chemical-grafted ACFM can capture both electron and hole carriers, accounting for inhibiting space charge accumulations and impeding charge transports in SiR-*g*-ACFM material. In particular, the electron trapping state of 0.6 eV below CBM originates completely from the benzoylphenyl of the grafted ACFM, indicating its competence for capturing and coupling hot electron carriers which can transform the electron transport (electronic kinetic energy) in host SiR into the molecular vibration (thermal energy) of the grafted ACFM, as shown by the right panel of Figure 3. Therefore, chemical-grafting ACFM renders a specific electronic property to release electronic kinetic energy into thermal energy for restricting hot electron production under a high electric field and thus improving dielectric breakdown strength of addition-cure SiR material [42,53].

The calculated band-gap of 3.7 eV for addition-cure SiR using a DFTB-based matsci-parameterized dispersion-correction approach represents a physically reasonable value that is consistent with high-level theoretical calculations for structurally analogous systems, while reflecting the distinct chemical composition of this crosslinked elastomer compared to polydimethylsiloxane (PDMS). The present band-gap value (3.7 eV) is consistent with hybrid functional calculations for analogous crosslinked siloxanes [54], with minor deviations from reference [19] attributable to methodological differences in exchange-correlation treatment and model specifications. Specifically, DFTB’s parameterized Hamiltonian and simplified integral scheme in our models yield marginally reduced electronic band-gaps compared to all-electron DFT adopted in reference [19]. These factors collectively explain the 0.5 eV discrepancy, which falls within the accepted methodological variance (−12%) for DFT-based predictions of polymer dielectrics. Our computed value for addition-cure SiR is 9.8% lower than the 4.1 eV experimental optical band-gap reported for PDMS, which is essentially attributed to their fundamental discrepancy in chemical structure [55]. Addition-cure SiR composed of vinyl-terminated polysiloxane with hydrosilicone oil crosslinkers contains Si-CH_2_-CH_2_-Si ethylene bridge bonds whose electron states lie within the Si-O skeletal band-gap and thus effectively narrow the fundamental electronic band-gap. Meanwhile, our calculated value avoiding excessive band-gap underestimation remarkably exceeds the theoretical minimum of 2.8 eV obtained from LDA calculations for all-anti-PDMS conformations [56]. Collectively, these considerations support the conclusion that 3.7 eV constitutes a robust theoretical estimate for electronic band-gaps of addition-cure SiR.

**Figure 4:**
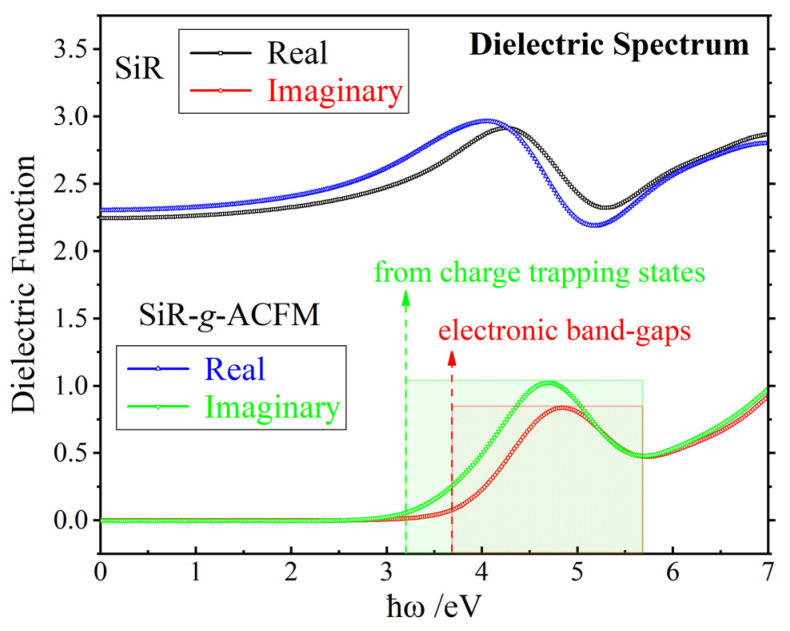
Dielectric functions of the SiR and SiR-*g*-ACFM bulk materials with a density of 1 g/cm^3^ as derived from the frequency-dependent polarizabilities of their precrosslink macromolecules.

Precrosslink macromolecules of addition-cure SiR represent the fundamental polymeric constituents and molecular compositions of the crosslinked SiR material and thus determine the dielectric function (polarizability frequency spectrum). The polarizability spectra of SiR and SiR-*g*-ACFM precrosslink macromolecules are calculated via first-principles methods and converted to dielectric functions of bulk materials at an aggregation density of 1 g/cm^3^, with results presented in Figure 4. In the low-frequency region below 3 eV, the real parts of the dielectric functions for both SiR and SiR-*g*-ACFM are constrained within the range of 2.5–2.7 and exhibit gradual increases with rising frequency, while the imaginary parts remain nearly zero. These characteristics align well with experimentally measured dielectric properties of addition-cure SiR materials reported in the literature [57,58]. When external polarization field frequency exceeds approximately 4 eV (corresponding to electronic energy band-gaps), the electronic resonant polarization and excitation (transitions to CBM) between the carbon–hydrogen (C-H) σ bonds of methyl (-CH_3_) or methylene (-CH_2_-) groups near VBM and the conjugate π* antibonding states of phenyl groups near CBM in addition-cure SiR macromolecules induce the strong polarization (real part) and absorption (imaginary part) peaks in dielectric functions of both SiR and SiR-*g*-ACFM materials within 3.5–5 eV and 4–5.5 eV frequency bands, respectively.

Compared to pristine SiR, the overall electronic displacement polarization arising from multiple strong dipoles on the grafted ACFM in SiR-*g*-ACFM results in an approximately 5% increase in the real part of the dielectric function across the 0–4 eV range. The frontier molecular orbitals contributed by the grafted ACFM in SiR-*g*-ACFM are positioned immediately above the VBM (occupied electron-bound states serving as hole traps) and below CBM (unoccupied electron-bound states serving as electron traps), as indicated in Figure 3. Therefore, the electronic transitions between these trap states, as well as between these states and the C-H σ bonds or conjugate π* antibonding states of the host SiR, lead to significantly enhanced absorption peaks in the imaginary part of the dielectric function for SiR-*g*-ACFM within the 3.7–5.7 eV frequency band, with an extension toward lower frequencies by approximately 0.5 eV (corresponding to charge trap depth), as shown in Figure 4. In summary, the multiple polar groups within the grafted ACFM provide abundant strong dipoles and introduce multilevel charge traps into addition-cure SiR.

### 3.2. Dipole Moment and Thermodynamic Miscibility with O_2_

**Figure 5:**
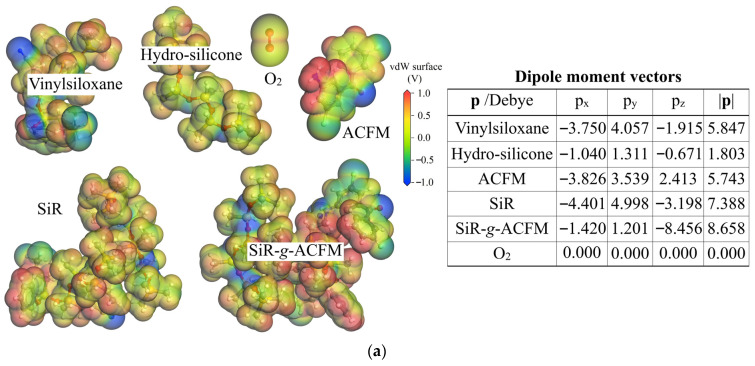

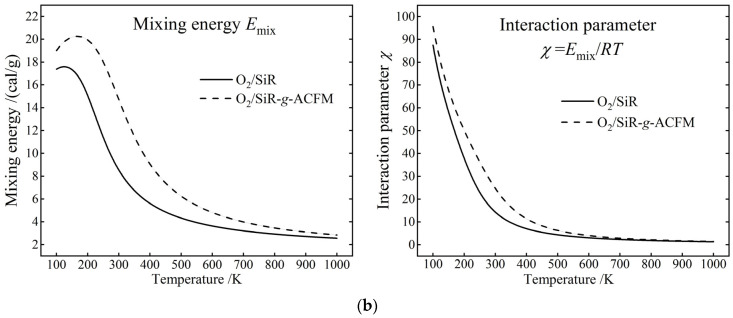
Blend compatibility of SiR and SiR-*g*-ACFM macromolecules with O_2_: (**a**) electrostatic potentials (distribution on vdW surface contoured by colors) and dipole moment magnitudes of SiR constituents (hydro-silicone and vinylsiloxane in *D*p = 6) and ACFM molecule as well as SiR and SiR-*g*-ACFM basic macromolecules (*D*p = 12); (**b**) mixing energies (left panel) and interaction parameters (right panel) of O_2_/SiR and O_2_/SiR-*g*-ACFM binary mixtures.

Dipole interaction with O_2_ is evaluated by the electrostatic potential and dipole moment of SiR or SiR-*g*-ACFM basic macromolecules from first-principles calculations, which accounts for molecular miscibility (blend compatibility) with O_2_, as illustrated in Figure 5. In comparison, the ACFM molecule possesses a remarkably greater and slightly smaller dipole moment magnitude (|**p**| = *μ*_d_) than that of hydro-silicone and vinylsiloxane constituents (*D*p = 6) respectively, leading to an evident *μ*_d_ improvement of SiR basic macromolecules (*D*p = 12) and a greater dipole difference from O_2_ molecules, as shown in Figure 5a. The SiR-*g*-ACFM basic macromolecule represents a considerably lower *μ*_d_ than the *μ*_d_ sum of the ACFM molecule and SiR basic macromolecule, which is attributed to the chemical grafting of the high-electrostatic-potential amino group (-NH_2_) of the ACFM molecule to the similar high-electrostatic-potential region of the SiR backbone, partially canceling the host dipole moment derived from the low electrostatic potential of the branched cyano group.

Interactions between polar molecules are primarily governed by electrostatic forces between their electrostatic dipole moments. In thermodynamics, blend compatibility between two polar molecules depends on the difference of their electrostatic dipole moments. A greater difference in dipole moment magnitudes of two mixing polar molecules results in a higher mixing energy or interaction parameter of their binary mixture under thermodynamic equilibrium, implying their poorer blend compatibility. Consistently, the chemical modification of grafting ACFM molecules provides a significantly stronger dipole moment in host SiR macromolecules, leading to the evident reduction in blend compatibility of SiR macromolecules with O_2_, as indicated by the higher mixing energy and interaction parameter of the O_2_/SiR-*g*-ACFM mixture compared to the O_2_/SiR mixture in Figure 5b.

### 3.3. Free Volume and O_2_ Uptake–Diffusion

**Figure 6:**
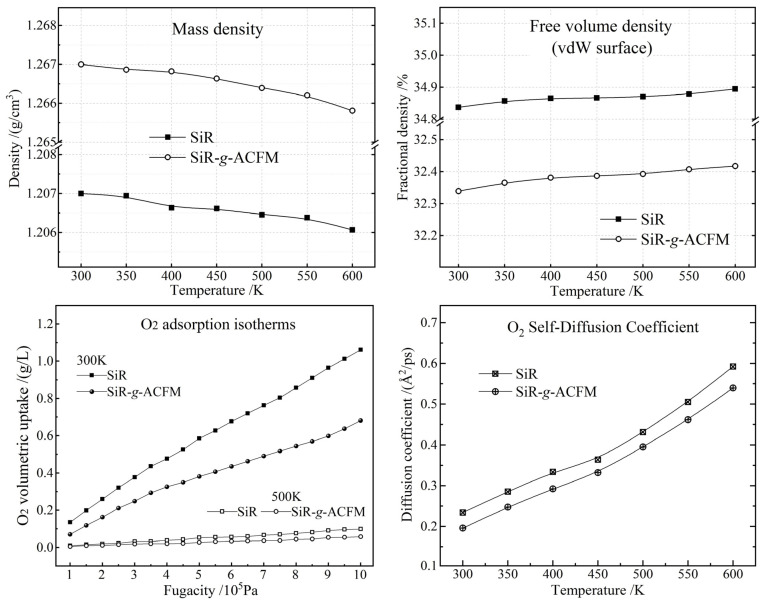
MD and MC simulation results of SiR and SiR-*g*-ACFM crosslinked materials: mass density (**top-left panel**), FFV (**top-right panel**), O_2_ adsorption isotherms (**bottom-left panel**) and O_2_ self-diffusion coefficients (**bottom-right panel**).

Fractional free volume (FFV) and mass density characterize the void spaces and interactions between polymer chains depending on molecular configurations in a condensed state in polymer materials, which are highly relevant to free-volume breakdown as well as O_2_ uptake and diffusion in the SiR and SiR-*g*-ACFM crosslinked materials, as shown in Figure 6. It is indicated from the MD simulated material models that ACFM grafts can increase mass density and decrease vdW-surface FFV of the SiR crosslinked material, acquiring denser aggregations due to stronger dipole interactions between polymer chains in the SiR-*g*-ACFM crosslinked material. Therefore, the ACFM graft facilitates condensations of the SiR polymer chains to holistically reduce free-volume density, concerting ACFM’s poorer molecular compatibility with O_2_ to decrease O_2_ adsorption uptakes even when the temperature rises up to 600 K, as indicated in the bottom-left panel of Figure 6. These findings substantiate that ACFM grafting orchestrates a synergistic suppression of free-volume-mediated dielectric breakdown and oxygen-related impurity levels in addition-cure SiR, thereby affording a robust strategy for next-generation high-field insulation.

Relative to the neat SiR, SiR-*g*-ACFM exhibits a markedly retarded O_2_ self-diffusion coefficient across the entire 300–600 K window (Figure 6, right panels). This deceleration is synergistically orchestrated by two graft-induced modifications: (i) a reduced FFV that constricts the accessible diffusion channels and amplifies steric hindrance, and (ii) a diminished O_2_/polymer compatibility that elevates the van der Waals friction along the polymer-chain surfaces. Collectively, the ACFM-mediated suppression of both free volume and polymer-penetrant interactions imposes a dual-barrier scenario, effectively immobilizing O_2_ infiltration in the addition-cure SiR matrix.

Throughout the 300–600 K window, SiR-*g*-ACFM manifests markedly superior resistance to O_2_ infiltration relative to its neat counterpart, as evidenced by suppressed thermodynamic uptake and retarded self-diffusion coefficients (bottom panels in Figure 6). This enhancement originates from a synergistic dipolar shield erected by the grafted ACFM: each benzophenone or amino/chloro/fluoro-benzoylphenyl moiety introduces a stronger local dipole than the siloxane backbone, generating an extended network of dipole interactions that simultaneously (i) shrinks the free volume and (ii) raises the chemical potential of dissolved O_2_. Consequently, the grafted architecture functions as a nanoscale dielectric barrier, intensifying polymer-chain coalescence and restraining oxygen penetration in the crosslinked SiR-*g*-ACFM material.

### 3.4. Thermal Stability

**Figure 7:**
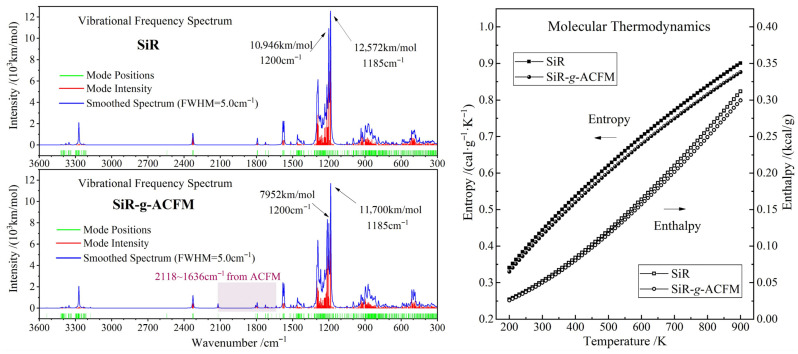
Molecular vibration frequency spectra (**left panel**) and thermodynamic properties (**right panel**) of SiR and SiR-*g*-ACFM precrosslink macromolecules.

Molecular vibration normal modes (intrinsic frequencies and intensities) and thermodynamic properties (entropy and enthalpy) in the geometrically optimized precrosslink macrostructures of SiR and SiR-*g*-ACFM are calculated by first-principles Hessian and 2-point difference of analytic forces to evaluate their thermal stabilities, as shown in Figure 7. The dominant Si-O stretching modes of 1200 cm^−1^ and 1185 cm^−1^ losing 27% and 7% of the integrated vibration intensities respectively from 10,946 km/mol and 12,572 km/mol to 7952 km/mol and 11,700 km/mol evidence the attenuation of Si-O backbone oscillator strength upon grafting ACFM, which indicates the decreased anharmonic coupling and delocalization, alleviating the earlier thermal scission under fluctuational heating. No considerable frequency shift is observed, implying the Si-O force constant is essentially preserved. Meanwhile, manifold minimal peaks (2118–1636 cm^−1^, <500 km/mol) arise solely in SiR-*g*-ACFM precrosslink macromolecules, deriving from the weak out-of-plane and wagging vibrations of the grafted ACFM moiety. These results suggest that the chemically grafted ACFM provides additional phonon channels for vibrational energy dissipation, which can effectively lower local temperature spikes that seed dielectric breakdown in SiR-*g*-ACFM material.

SiR-*g*-ACFM exhibits marginally lower molecular entropy and enthalpy than neat SiR, as shown by the right panel in Figure 7. The lower entropy (Δ*S* < 0) indicates a stiffer local structure around the grafted ACFM, while the lower enthalpy (Δ*H* < 0) implies exothermic grafting and hereby a more stable macrostructure relevant to thermoelectric breakdown or aging. Lower enthalpy density and extra dissipation pathways delay the critical build-up of vibrational energy density along the SiR backbone, retarding the onset of thermal runaway to higher fields or temperatures. The grafted ACFM segments occupy and rigidify interchain voids, reducing both free-volume density and its fluctuation amplitude, which can inhibit electron avalanches that initiate free-volume breakdown and hereby raise intrinsic dielectric strength for the SiR material.

**Figure 8:**
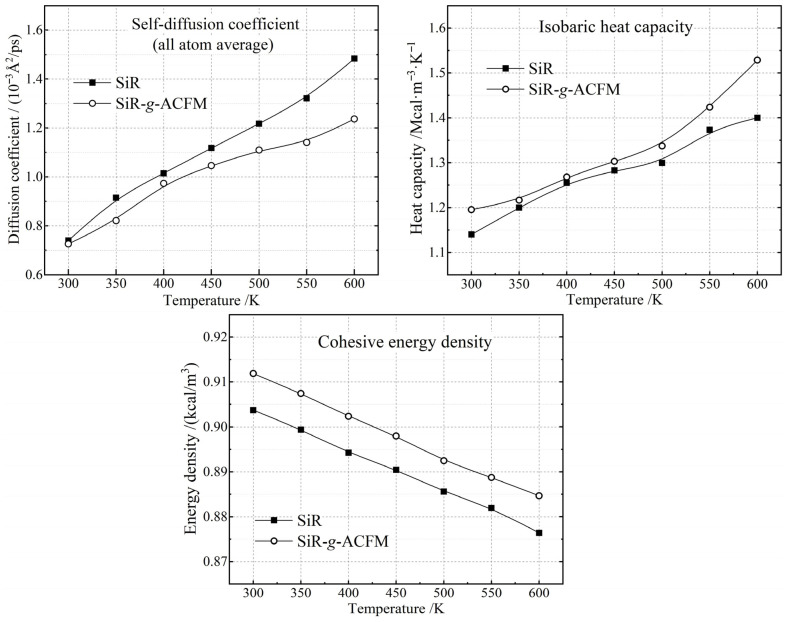
Atom-averaged self-diffusion coefficients (**top-left panel**), isobaric heat capacities (**top-right panel**), and cohesive energy densities (**bottom panel**) of SiR and SiR-g-ACFM crosslinked materials at 300–600 K.

From each moving atom and statistical average of MD fluctuation in SiR and SiR-*g*-ACFM crosslinked materials at various temperatures, it is effective to evaluate thermal motions of SiR polymer chains by the atom-averaged self-diffusion coefficient and isobaric heat capacity. As shown in the left panel of Figure 8, dipole interactions from ACFM polar groups result in lower atom-averaged self-diffusion coefficients even when the temperature rises to 600 K, as a manifestation of ACFM grafts restraining thermal motions of SiR polymer chains consistent with the holistically increased mass density and decreased FFV. Meanwhile, the ACFM graft increases isobaric heat capacity of addition-cure SiR material in a comprehensive 300–600 K range, as illustrated in the right panel of Figure 8, which will alleviate the local thermal shock that initiates thermoelectric breakdown above the operating temperature in power transmission systems.

It is important to contextualize these thermal stability results within the operational temperature regime of cable accessories. The glass transition temperature (*T*_g_) of high-molar-mass silicone rubber is experimentally established at approximately 150 K (−123 °C) for systems with number-average molar mass (*M*_n_) higher than 4 kg/mol, where *T*_g_ becomes independent of molecular weight and ring size [59]. Our modeled precrosslink macromolecules (*M*_n_ = 7.5 kg/mol and 8.0 kg/mol for SiR and SiR-*g*-ACFM respectively) and crosslinked polymer molecules (45 and 48 kg/mol respectively) both exceed this threshold, ensuring that the base *T*_g_ remains constant at ~150 K regardless of chain length or grafting. Our simulation temperature range (300–600 K) lies ~150 K above *T*_g_, deep within the rubbery plateau where mechanical compliance is governed by entropic elasticity. Even with conservative estimates of *T*_g_ elevation (20–30 K) due to ACFM grafting, a perturbation smaller than the approximately 90 K difference between our *M*_n_ (7.5–8 kg/mol) and the low-molar-mass regime (<4 kg/mol) where *T*_g_ increases abnormally due to crystallization, the SiR-*g*-ACFM at 300 K remains >120 K above *T*_g_, ensuring preserved flexibility for cable accessory applications (typical operating range: −50–200 °C). This is corroborated by the maintained atomic self-diffusion coefficients (Figure 8) and chain segmental mobility evidenced in MD trajectories.

The mechanical integrity of SiR-*g*-ACFM is further evaluated through CED analysis (bottom panel of Figure 8). Throughout the 300–600 K range, SiR-*g*-ACFM exhibits moderately elevated CED compared to neat SiR (0.904–0.876 kcal/m^3^ vs. 0.912–0.885 kcal/m^3^, an average 0.86% increase), indicating enhanced intermolecular cohesion and improved resistance to mechanical deformation. Importantly, this modest CED increase occurs deep within the rubbery plateau, ensuring that the SiR material retains the low Young’s modulus and high elastic recovery required for cable accessory flexibility and installation.

### 3.5. Oxidation Stability

**Figure 9:**
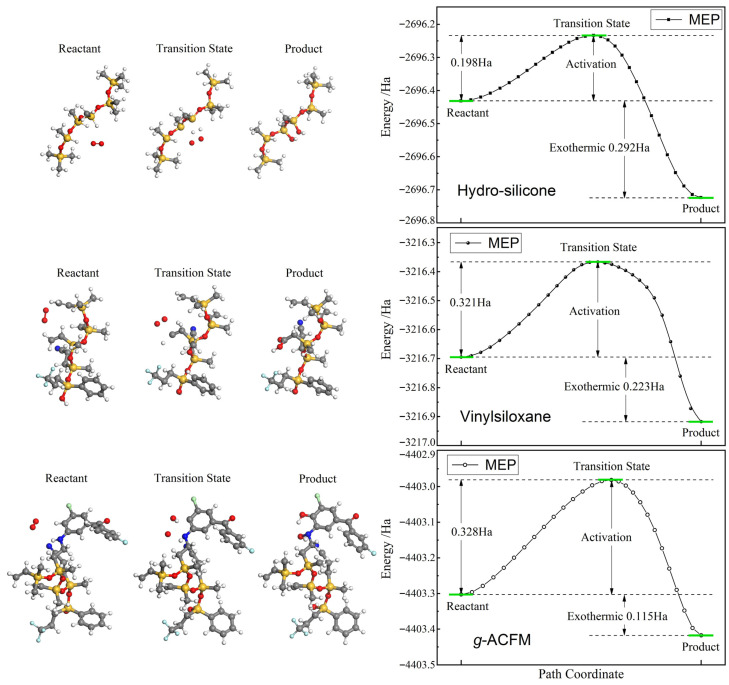
Schematics and minimum energy paths (MEPs) in chemical reactions of hydro-silicone -Si-H, vinylsiloxane -CH=CH_2_, and *g*-ACFM -NH/-CH with O_2_, producing -Si-OH, -CH_2_-COOH, and -N-OH/-C-OH respectively. The white, gray, blue, red, light blue, orange and light green spheres symbolize hydrogen, carbon, nitrogen, oxygen, fluorine, silicon and chlorine bonding atoms respectively.

In the chemical components of addition-cure SiR, being residual from addition or crosslink reactions, the silicone hydride (-Si-H) of the hydro-silicone constituent and the vinyl (-CH=CH_2_) of the vinylsiloxane constituent are most susceptible to oxidization by O_2_ in special conditions such as high temperature or ultraviolet irradiation in the presence of oxygen. During the oxidation process of addition-cure SiR, one oxygen molecule reacts with two adjacent hydro-silicone -Si-H, producing two silanol groups (-Si-OH), or with one vinylsiloxane -CH=CH_2_, producing preferably one carboxyethyl (-CH_2_-COOH) rather than two formaldehydes (CH_2_=O), as verified by their reaction pathways from our first-principles calculations. In the grafted ACFM (*g*-ACFM) of SiR-*g*-ACFM macromolecules, a nitrogen–hydrogen bond (-NH) left after grafting presents the most reactive component with O_2_ to produce a nitrogen-hydoxyl (-N-OH). Furthermore, as derived from our first-principles calculations on targeting the lowest-energy oxidation byproduct, given one oxygen atom in O_2_ reacts primarily with -NH in *g*-ACFM, the other oxygen atom reacts with the adjacent carbon–hydrogen bond (-CH) of chlorophenyl (-C_6_H_3_Cl-) into a chlorophenol group (-C_6_H_2_OHCl-), as shown in the left panel of Figure 9.

Oxidative stability of a polymer is generally evaluated by the energy barrier and released energy in oxidation reactions of its polymerizing constituents and any chemical modifier. A higher reaction barrier (activation energy) or lower released energy (exothermicity) in an oxidation process indicates a stronger oxidative stability. The activation energy and exothermicity during the oxidation of addition-cure SiR constituents (hydro-silicone/vinylsiloxane) calculated with their simplified models (*D*p = 6) are 0.198/0.321 Ha and 0.292/0.223 Ha respectively, as indicated in the right panel of Figure 9. By contrast, the activation energy and exothermicity for the grafted ACFM (*g*-ACFM) are 0.328 Ha and 0.115 Ha respectively. The grafting agent exhibits a higher energy barrier and a lower released energy in the oxidation process compared to both the two constituents of addition-cure SiR. The harder chemical reaction with O_2_ of *g*-ACFM demonstrates its preferable resistance to oxidation compared to SiR polymer chains, thereby achieving the overall enhanced oxidative stability in SiR-*g*-ACFM macromolecules. This finding is crucial for addition-cure SiR material being exposed to oxidative environments and requiring long-term stability and reliability.

## 4. Conclusions

Chemical modification of grafting voltage stabilizer ACFM onto addition-cure SiR is systematically investigated through a comprehensive set of first-principles calculations, Monte Carlo simulations, and molecular dynamics simulations, focusing on charge traps, oxygen uptake/diffusion, thermal stability, and oxidative stability. First-principles calculations reveal that both the hole and electron shallow traps can be introduced by grafting ACFM into addition-cure SiR, being suggested for suppressing space charge accumulation and electron avalanche breakdown. ACFM grafting is verified to decrease molecular compatibility with O_2_ and intensify polymer coalescence in addition-cure SiR material due to the introduced multiple dipoles, leading to substantial reductions in O_2_ uptake and diffusion even above 500 K, which implies improved resistances to oxygen permeation and free-volume dielectric breakdown. Moreover, ACFM grafting restricts thermal motions of the SiR polymer chains and increases heat capacity throughout the 300–600 K temperature range, implying enhanced insulation strength against thermal–electric breakdown. It is eventually predicted through oxidation path calculations that the grafting agent favors the oxidative stability of addition-cure SiR. The present research highlights the significant potential of voltage stabilizer graft modification for high-temperature insulation performance and oxidative degradation of addition-cure SiR, offering valuable insights for developing advanced polymer dielectrics in outdoor and automotive applications under harsh environmental conditions.

## Figures and Tables

**Table 1:** Methodology in first-principles calculations and MC molecular simulations of SiR and SiR-*g*-ACFM macromolecules.

First-Principles Calculation (DFTB+)	Schemes	Specification
Electronic Hamiltonization	Slater–Koster parameter set [46]	matsci atomic parameters
	Self-consistent charges [47]	1 × 10^−8^ Ha/atom (1 Ha = 27.2 eV)
	Eigensolver	Divide and conquer
	Broyden charge mixing [48]	Mixing amplitude = 0.2
	Orbital occupancy smearing [49]	Distribution function: Methfessel–Paxton; Amplitude = 0.003 Ha
Non-covalent interaction	Dispersion correction	Tkatchenko–Scheffler
Geometry optimization	Convergence tolerance	Energy: 0.02 kcal/mol; Force: 0.1 kcal/mol/Å; Displacement: 0.001 Å
MC molecular simulation (**Blends**)	Schemes	Specification
Energy	Force field and charges	PCFF [50]
O_2_/SiR and O_2_/SiR-*g*-ACFM mixtures	Blend thermodynamics	Energy/cluster samples: 10^7^/10^5^; Energy bin width: 0.02 kcal/mol; Iterations: 20; Reference temperature: 298 K

## Data Availability

The theoretical methods and results are available from the corresponding author on request.
